# Sex-Associated Differences in Short-Term Outcomes in Patients with Deep Sternal Wound Infection after Open-Heart Surgery

**DOI:** 10.3390/jcm11247510

**Published:** 2022-12-19

**Authors:** Ihor Krasivskyi, Borko Ivanov, Kaveh Eghbalzadeh, Frederike Fehlau, Stephen Gerfer, Clara Großmann, Ahmed Elderia, Anton Sabashnikov, Parwis Baradaran Rahmanian, Navid Mader, Ilija Djordjevic, Thorsten Wahlers

**Affiliations:** 1 Department of Cardiothoracic Surgery, University Hospital Cologne, 50937 Cologne, Germany; 2 Department of Cardiothoracic Surgery, Helios Hospital Siegburg, 53721 Siegburg, Germany

**Keywords:** deep sternal wound infection, cardiac surgery, sex, VAC therapy

## Abstract

Deep sternal wound infection (DSWI) is a feared complication after cardiac surgery. The impact of sex-related differences on wound infection prevalence is poorly understood. Our aim was to evaluate the effect of sex on short-term outcomes in patients with DSWI after open-heart surgery. The study was a retrospective cohort study. A total of 217 patients with DSWI were identified and retrospectively analyzed using our institutional database. Patients were divided into two groups: males (*n* = 150) and females (*n* = 67). This study also includes a propensity score based matching (PSM) analysis (male group (*n* = 62) and female group (*n* = 62)) to examine the unequal groups. Mean age (*p* = 0.088) and mean body mass index (BMI) (*p* = 0.905) did not significantly differ between both groups. Vacuum assisted closure (VAC) therapy was performed among most patients (82.3% (male group) vs. 83.9% (female group), *p* = 0.432). The most commonly isolated bacteria from the wounds were Staphylococcus epidermidis and Staphylococcus aureus in both groups. Acute renal failure was significantly higher (*p* = 0.010) in the male group compared to the female group. However, dialysis rate did not significantly differ (*p* = 0.491) between male and female groups. Further secondary outcomes showed no major differences between the groups. Likewise, in-hospital mortality rate did not differ significantly (*p* = 0.680) between both groups. Based on our data, sex has no impact on deep wound infection prevalence after cardiac surgery.

## 1. Introduction

Deep sternal wound infection (DSWI) is one among several serious complications after open-heart surgery [1]. This feared complication after median sternotomy with an incidence between 0.5% and 6.8% is associated with prolonged hospital stay and higher mortality [2]. Moreover, antibiotic therapy and additional surgical operations affect healthcare costs immensely [3]. 

Despite the use of modern skin antiseptic solution the surgical area still remains contaminated [4]. The in-hospital mortality rate differs between 7% and 35% in patients with a wound infection after open-heart surgery [2,5]. Therefore, the prevention of DSWI is one of the most important goals in cardiac surgery [6]. 

Fast healing of the wound area is the primary aim of the therapy performed [7]. In this regard, vacuum assisted closure (VAC) therapy has been developed over the last decades to accelerate healing tendency [8]. Moreover, it leads to reduced mortality and is a first-line treatment for bridging to sternal reconstruction [9]. 

The use of both internal thoracic arteries, excessive use of electrocoagulation and bone wax, diabetes mellitus, long cardio-pulmonary bypass (CPB) time, reoperation, and prolonged mechanical ventilation are associated with a higher incidence of DSWI after cardiac surgery [10,11]. However, differences between male and female patients with DSWI have not been fully examined in the last decades [12,13,14,15]. Thus, we aimed to investigate the sex-associated differences in short-term outcomes in patients with a deep sternal wound infection after open-heart surgery.

## 2. Materials and Methods

**Figure 1:**
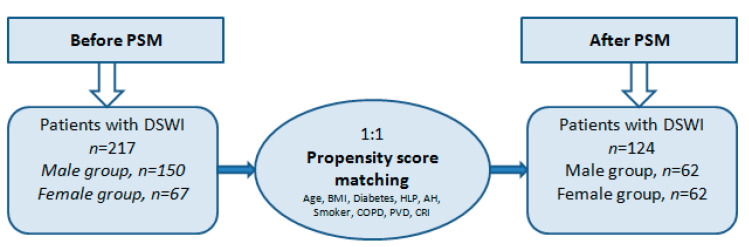
Sample distribution with DSWI before and after PSM. PSM, propensity score matching; DSWI, deep sternal wound infection; BMI, body mass index; HLP, Hyperlipidaemia; AH, arterial hypertension; COPD, chronic obstructive pulmonary disease; PVD, peripheral vascular disease; CRI, chronic renal insufficiency.

**Figure 2:**
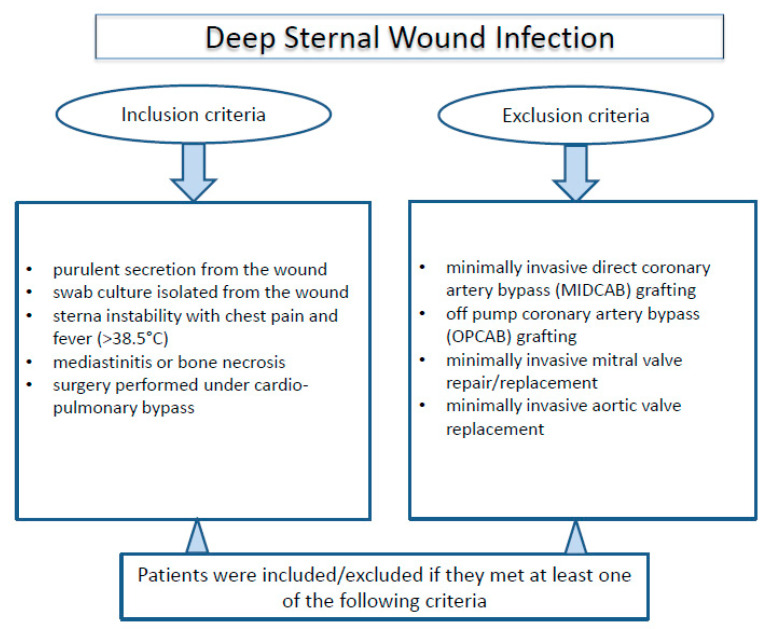
Inclusion and exclusion criteria.

This research is a retrospective single center cohort study. All patients who developed sternal wound infection at the University hospital Cologne from June 2011 to October 2019 after open-heart surgery were included. For the analysis, all patients were divided into two sex categories: male group (*n* = 150) and female group (*n* = 67). This study also includes a propensity score based matching (PSM) analysis (male group (*n* = 62) and female group (*n* = 62)) to examine the unequal groups (Figure 1). Patients who had at least one from the down listed criteria were included in the study (Figure 2):

- Purulent secretion from the wound.
- Swab culture isolated from the wound.
- Sternal instability with chest pain and fever (>38.5 °C).
- Mediastinitis or bone necrosis.
- Surgery performed under cardio-pulmonary bypass.

Whereas, patients, who underwent at least one of the following criteria, were excluded from the study:

- Minimally invasive direct coronary artery bypass (MIDCAB) grafting.
- Off pump coronary artery bypass (OPCAB) grafting.
- Minimally invasive mitral valve repair/replacement.
- Minimally invasive aortic valve replacement.

### 2.1. Data Collection 

Preoperative, operative, and postoperative data were withdrawn from the database, which belongs to the mandatory German Cardiac Surgery Quality Assurance System. All perioperative data were gathered during the patient’s hospital stay and analyzed retrospectively.

### 2.2. Outcome Analysis 

Our research primary outcome was in-hospital mortality after cardiac surgery. Acute kidney injury, dialysis, duration of mechanical ventilation, tracheotomy rate, intensive care unit (ICU) and hospital stay were secondary outcomes. 

### 2.3. Ethics

This research was conducted under the Declaration of Helsinki (as revised in 2013). According to the Ethics Committee of the Medical Faculty of the University of Cologne there is no need to apply for ethical approval as under the German law retrospective clinical studies can be conducted without separate ethical approval from the local ethics committee.

### 2.4. Statistical Methods 

Statistical analysis was conducted using the Student *t*-Test (for normally distributed continuous variables) or Mann–Whitney-U test (for not normally distributed continuous variables). Categorical variables were analyzed using the Chi-square test. We presented continuous variables as mean ± standard deviation (SD), while categorical variables were shown as a percentage of the sample. We used Fisher exact test when the minimum expected count of cells was <5. Moreover, we ran the logistical regression in order to create the predicted variable. The 1:1 PSM was used with a 0.2 caliper set. The sample size was calculated using the power of 80%, two-tailed test of the null hypothesis with alfa (=0.05) and a value of beta (=0.10) as the first and second type errors. In the study the *p*-value of <0.05 was defined as significant. Statistical analysis was performed using Statistical Package for Social Sciences, version 28.1 (SPSS Inc., Chicago, IL, USA).

## 3. Results

### 3.1. Baseline and Preoperative Data

Table 1 summarizes patient demographic and preoperative risk factors. The mean BMI of the study population was 28.6 ± 5.0 (male group) vs. 29.0 ± 7.4 (female group) kg/m^2^, *p* = 0.007 before PSM and 29.7 ± 5.2 (male group) vs. 29.4 ± 7.3 (female group) kg/m^2^, *p* = 0.905 after PSM. Diabetes did not differ between both groups (*n* = 66 (44.0%) male patients vs. *n* = 36 (53.7%) female patients, *p* = 0.293 before PSM and *n* = 32 (51.6%) male patients vs. *n* = 33 (53.2%) female patients, *p* = 0.857). Renal insufficiency was found in 40 (26.7%) male vs. 15 (22.7%) female patients, *p* = 0.274 before PSM and 16 (25.8%) male vs. 15 (24.2%) female patients, *p* = 0.597. Further data did not significantly differ between both groups. 

### 3.2. Intraoperative Data

Table 2 shows all relevant intraoperative data. The majority of patients underwent bypass surgery (*n* = 111 (74.0%) male group vs. *n* = 40 (59.7%) female group, *p* = 0.362 before PSM and *n* = 44 (71.0%) male group vs. *n* = 37 (59.7%) female group, *p* = 0.186, after PSM). Use of both internal thoracic arteries was significantly higher (*p* = 0.023) in the male group (*n* = 53 (35.3%)) compared to the female group (*n* = 14 (20.9%)) before PSM. However, after PSM the use of both ITAs did not significantly differ (*p* = 0.832) between both groups. Cardiopulmonary bypass time and cross clamp time were measured by 96.4 ± 60.3 (male group) vs. 96.5 ± 48.3 (female group) minutes (*p* = 0.193) and 57.6 ± 41.7 (male group) vs. 60.0 ± 32.6 (female group) minutes (*p* = 0.425), respectively, before PSM. Likewise, CPB time (*p* = 0.505) and cross clamp time (0.749) did not significantly differ between the above-mentioned groups after PSM. 

### 3.3. Wound Revision Data

**Figure 3:**
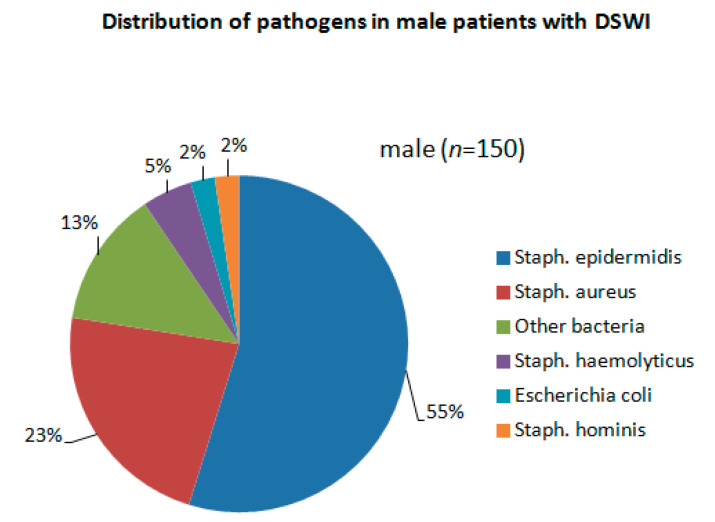
Distribution of pathogens in male patients with DSWI.

**Figure 4:**
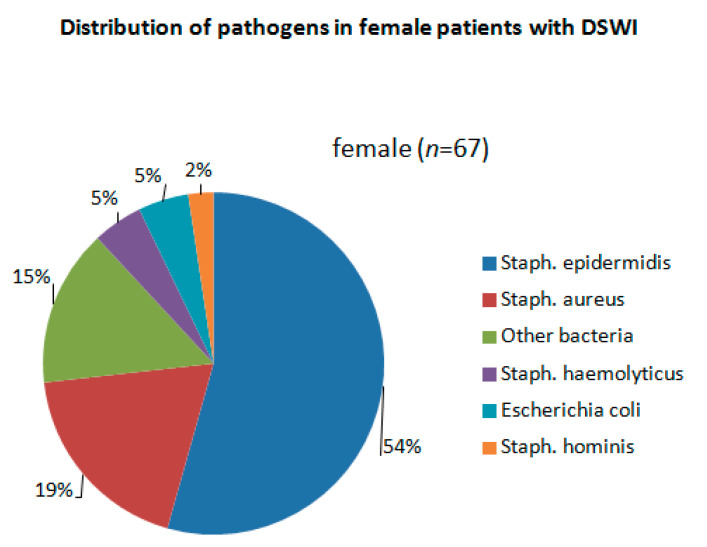
Distribution of pathogens in female patients with DSWI.

Table 3 summarizes all relevant wound revision data. VAC therapy was performed among most patients (76.6% (male group) vs. 85.2% (female group), *p* = 0.188 before PSM and 82.3% (male group) vs. 83.9 (female group), *p* = 0.432 after PSM). In contrast, direct wound closure was conducted only in 32 (23.4%) male vs. 9 (14.8%) female patients, *p* = 0.116 before PSM and 11 (17.7%) male vs. 10 (16.1%) female patients, *p* = 0.125 after PSM. The most commonly isolated bacteria from the wounds were Staphylococcus epidermidis and Staphylococcus aureus in both groups (Figure 3 and Figure 4). Further wound revision parameters did not significantly differ between both groups. 

### 3.4. Primary and Secondary Outcomes

Primary and secondary outcomes are shown in Table 4. Acute renal failure was significantly higher in the male group compared to the female group before PSM (*p* = 0.013) and after PSM (*p* = 0.010). However, dialysis rate after the operation did not significantly differ between the male (*n* = 8 (5.4%)) and the female groups (*n* = 5 (7.6%)), *p* = 0.547 before PSM and (*n* = 3 (4.8%)) and the female groups (*n* = 5 (8.2%)), *p* = 0.491) after PSM. The duration of mechanical ventilation (*p* = 0.005) and the tracheotomy rate (*p* = 0.033) were significantly higher in the male group compared to the female group before PSM. However, the above-mentioned data (mechanical ventilation (*p* = 0.527) and the tracheotomy rate (*p* = 0.496) did not significantly differ between both groups after PSM. The mean length of ICU stay was significantly higher (*p* = 0.004) in the male group (7 ± 9 days) compared with the female group (5 ± 4 days) before PSM. However, the mean length of ICU stay did not differ between both groups (6 ± 7 days in the male group vs. 4 ± 3 days in the female group, *p* = 0.179) after PSM. The in-hospital mortality rate did not significantly differ between two groups before (*p* = 0.622) and after (*p* = 0.680) PSM.

## 4. Discussion

In our study, we investigated sex-associated differences in short-term outcomes in patients with DSWI after open-heart surgery. Our results showed that sex has no impact on DSWI after cardiac surgery.

Gender-related factors that simplify the bacterial penetration of the wound or reduce immune response are of interest in the current literature [16]. Sex-specific reports of outcomes after cardiac surgery have been controversially discussed [17,18]. Generally, women who underwent coronary artery bypass grafting (CABG) procedures have more comorbidities compared to men [18,19]. Furthermore, women undergo more commonly emergent surgical procedures [20,21]. Moreover, coronary revascularization is less often and significantly later performed in females due to protective hormone levels in the premenopausal period [22,23]. In contrast, the male gender is associated with lower left ventricular ejection fraction, has 3-vessel disease significantly more often, and receives more arterial grafts compared to the female gender [22,24]. Likewise, men undergo significantly more frequent CABG reoperation [25]. 

However, there is a lack of studies that investigated the impact of sex differences on short-, mid- and long-term outcomes of patients with DSWI after cardiac surgery [17,26,27]. Several studies showed that the female sex is associated with a higher risk of DSWI after CABG surgery compared to men [17,23,28]. Itagaki et al. [29] stated that the female gender was an independent risk factor for DSWI development in the postoperative period [29]. In addition, Gatti et al. [17] analyzed 2.872 patients after isolated CABG surgery and showed that the female sex was a strong predictor of DSWI. However, the use of both internal thoracic arteries was routinely performed in 70% of all cases [17]. In this regard, harvesting of both internal thoracic arteries (ITA) is associated with a decrease in tissue blood supply [30]. Thus, the utilization of both major arteries of the chest could lead to local hypoperfusion and might be associated with a higher risk of developing DSWI [31]. We found significantly lower (*p* = 0.023) use of bilateral ITA grafts in the female group compared to the male group before PSM. However, there were no statistical differences (*p* = 0.832) regarding use of bilateral ITA grafts between both groups after PSM. The lack of statistical difference might be misleading as the numbers after PSM are small. Therefore, this difference might have caused lower numbers of infections in the female group. 

Further authors found that women with diabetes presented an increased risk for DSWI after bypass surgery [32]. Moreover, women with diabetes showed significantly higher risk for mediastinitis after open-heart surgery then men [33]. Furthermore, several studies mentioned that old female patients with obesity have a significantly more often DSWI after cardiac surgery compared to male patients [34,35]. Moreover, the combination of diabetes mellitus and obesity in female patients significantly increases the risk for DSWI after cardiac surgery [17]. Therefore, various authors do not suggest using both ITA grafts for women, especially those with diabetes and obesity [36,37]. 

Furthermore, Copeland et al. [38] stated that women with medium and large breast sizes have an increased risk of DSWI after open-heart surgery. The authors hypothesized that the weight of large breasts could produce inferolateral tension on the midline sternotomy wound and could contribute to the dehiscence of the wound [38]. In our study, we did not include breast size parameters due to the data sensibility. Therefore, we could not provide any statement regarding the above-mentioned parameter. 

Postoperative wound infection with mediastinitis could significantly prolong the length of in-hospital stay [39,40]. The immense costs of critical medical care in those cases may be exorbitant [3,11,40]. In our study, we found that the male group showed significantly higher (*p* = 0.004) ICU stay compared to the female group before PSM. However, the average ICU (0.179) and in-hospital stay did not significantly (*p* = 0.093) differ between both groups after PSM. 

DSWI prevalence after open-heart surgery remains high despite enhancement in perioperative management [41]. However, we did not find significant differences (*p* = 0.680) in the mortality rate between males and females. It could be explained by the fact that we used VAC therapy, allowing for the immediate drainage of purulent fluid and providing the optimal conditions for secondary closure [8]. 

Several authors found higher rates of end-organ failure in men compared to women [42,43]. Likewise, the acute renal failure rate was significantly higher (*p* = 0.010) in the male group in our study. Several studies mentioned that the protective role of the female sex in the development of acute renal failure could be explained by the effects of sex hormones on cellular processes in the pathogenesis of acute renal failure [43,44]. Nevertheless, the dialysis rate did not significantly (*p* = 0.491) differ between both groups. 

Based on our findings there were no bacterial culture differences isolated from the wound among both groups. It could lead to the assumption that bacterial causative pathogens do not have sex preferences.

## 5. Conclusions

Our findings showed that sex has no impact on in-hospital mortality in patients with DSWI after cardiac surgery. Likewise, further secondary outcomes did not significantly differ between both groups. However, acute renal failure was significantly higher in the male group compared to the female group. 

## 6. Study Limitations

This research has a number of limitations. Firstly, a retrospective single-center analysis with a small patient cohort could be potentially showing a low statistical power. Secondly, our main focus was short-term outcomes, and we did not investigate long-term outcomes and quality of life measures. Thirdly, we did not evaluate specific pathophysiological conditions. 

## Figures and Tables

**Table 1:** Patient’s baseline preoperative demographics.

	before PSM			after PSM		
	Male (*n* = 150)	Female (*n* = 67)	*p*-Value	Male (*n* = 62)	Female (*n* = 62)	*p*-Value
Age (years), mean ± SD	63 ± 12	66 ± 12	0.606	64 ± 11	66 ± 13	0.088
BMI (kg/m^2^), mean ± SD	28.6 ± 5.0	29.0 ± 7.4	0.007	29.7 ± 5.2	29.4 ± 7.3	0.905
Diabetes, *n* (%)	66 (44.0%)	36 (53.7%)	0.293	32 (51.6%)	33 (53.2%)	0.857
Hyperlipidemia, *n* (%)	118 (78.7%)	58 (86.6%)	0.347	46 (74.2%)	53 (85.5%)	0.117
Arterial hypertension, *n* (%)	136 (90.7%)	62 (92.5%)	0.761	56 (90.3%)	57 (91.9%)	0.752
Smoker, *n* (%)	73 (48.7%)	25 (37.3%)	0.290	29 (46.8%)	22 (35.5%)	0.229
COPD, *n* (%)	31 (20.7%)	12 (17.9%)	0.882	15 (24.2%)	12 (19.4%)	0.218
PVD, *n* (%)	22 (14.8%)	16 (23.9%)	0.261	10 (16.1%)	15 (24.2%)	0.135
Renal insufficiency, *n* (%)	40 (26.7%)	15 (22.7%)	0.274	16 (25.8%)	15 (24.2%)	0.597

PVD, peripheral vascular disease; BMI, body mass index; COPD, chronic obstructive pulmonary disease; PSM, propensity score matching.

**Table 2:** Intraoperative data.

	before PSM			after PSM		
	Male (*n* = 150)	Female (*n* = 67)	*p*-Value	Male (*n* = 62)	Female (*n* = 62)	*p*-Value
CABG, *n* (%)	111 (74.0%)	40 (59.7%)	0.362	44 (71.0%)	37 (59.7%)	0.186
Heart valve surgery, *n* (%)	39 (26.0%)	27 (40.2%)	0.052	18 (29.0%)	25 (40.3%)	0.073
Urgent procedure, *n* (%)	30 (20.1%)	13 (19.4%)	0.769	10 (16.1%)	11 (17.7%)	0.811
CPR before surgery, *n* (%)	7 (4.7%)	0 (0.0%)	0.071	3 (4.8%)	0 (0.0%)	0.244
Reoperation, *n* (%)	9 (6.1%)	1 (1.5%)	0.126	2 (3.2%)	1 (1.6%)	0.346
Use of left ITA graft, *n* (%)	125 (83.3%)	53 (80.3%)	0.360	47 (75.8%)	49 (80.3%)	0.545
Use of both ITA graft, *n* (%)	53 (35.3%)	14 (20.9%)	0.023	15 (24.2%)	14 (22.6%)	0.832
Bone wax, *n* (%)	71 (48.3%)	26 (39.4%)	0.480	23 (37.1%)	25 (41.0%)	0.811
CPB time (min), mean ± SD	96.4 ± 60.3	96.5 ± 48.3	0.193	97 ± 52	99 ± 46	0.505
CC time (min), mean ± SD	57.6 ± 41.7	60.0 ± 32.6	0.425	58 ± 35	61 ± 33	0.749

CABG, coronary artery bypass grafting; CPB, cardiopulmonary bypass; ITA, internal thoracic artery; CPR, cardiopulmonary resuscitation; CC time, cross clamp time; PSM, propensity score matching; heart valve surgery: aortic valve replacement, mitral valve repair/replacement, tricuspid valve repair.

**Table 3:** Wound revision data.

	before PSM			after PSM		
	Male (*n* = 150)	Female (*n* = 67)	*p*-Value	Male (*n* = 62)	Female (*n* = 62)	*p*-Value
HbA1c, %, mean ± SD	6.8 ± 1.5	7.1 ± 1.4	0.686	6.5 ± 1.5	7.0 ± 1.4	0.667
CRP mg/L, mean ± SD	93.4 ± 85.8	94.4 ± 83.9	0.969	104 ± 90	97 ± 83	0.676
Leukocytes 10×9/L, mean ± SD	10.1 ± 4.4	10.0 ± 3.3	0.090	9.4 ± 3.8	9.4 ± 3.6	0.653
Wound secretion, n (%)	28 (20.4%)	10 (16.4%)	0.563	13 (22.4%)	9 (16.1%)	0.391
Sternal instability, n (%)	77(56.2%)	36 (59.0%)	0.417	29 (50.9%)	32 (57.1%)	0.504
Sternal wire removal, n (%)	72 (53.7%)	28 (46.7%)	0.225	38 (61.2%)	25 (40.3%)	0.032
Direct wound closure, n (%)	32 (23.4%)	9 (14.8%)	0.116	11 (17.7%)	10 (16.1%)	0.125
VAC therapy, n (%)	105 (76.6%)	52 (85.2%)	0.188	51 (82.3%)	52 (83.9%)	0.432
VAC therapy, days, median ± SD	17 ± 13	16 ± 14	0.265	18 ± 14	17 ± 15	0.324

CRP, C-reactive protein; VAC, vacuum-assisted closure; PSM, propensity score matching.

**Table 4:** Postoperative data.

	before PSM			after PSM		
	Male (*n* = 150)	Female (*n* = 67)	*p*-Value	Male (*n* = 62)	Female (*n* = 62)	*p*-Value
MV, hours, mean ± SD	90.3 ± 27.2	39.0 ± 53.7	0.005	35 ± 50	39 ± 55	0.527
Tracheotomy, n (%)	9 (6.1%)	0 (0.0%)	0.033	2 (3.25)	0 (0.0%)	0.496
Postoperative delirium, n (%)	36 (24.5%)	11 (16.9%)	0.221	16 (25.8%)	11 (18.3%)	0.386
Acute renal failure, n (%)	41 (27.7%)	8 (12.1%)	0.013	21 (33.9%)	8 (13.1%)	0.010
Dialysis, n (%)	8 (5.4%)	5 (7.6%)	0.547	3 (4.8%)	5 (8.2%)	0.491
ICU stay, days, mean ± SD	7 ± 9	5 ± 4	0.004	6 ± 7	4 ± 3	0.179
Hospital stay, days, mean ± SD	29 ± 13	20 ± 14	0.087	29 ± 12	19 ± 14	0.093
In-hospital mortality, n (%)	5 (3.4%)	2 (3.0%)	0.622	3 (4.8%)	2 (3.3%)	0.680

ICU, intensive care unit; CPR, cardiopulmonary resuscitation; MV, mechanical ventilation; PSM, propensity score matching.

## Data Availability

Data can be obtained on reasonable request.
